# Supplementary material for: Comparison of bacterial diversity and abundance between sexes of Leptocybe invasa Fisher & La Salle (Hymenoptera: Eulophidae) from China
Source: PeerJ. 2020 Jan 15;8:e8411. doi: 10.7717/peerj.8411 (PMC6969552; doi:10.7717/peerj.8411)
Supplement: Table S1 [file peerj-08-8411-s001.docx]

**Additional file1: Table S1: Basic information of the primers**

| Gene name | Primer name | 5’- 3’ |
| --- | --- | --- |
| COI | LCO1490 | GGTCAACAAATCATAAAGATATTGG |
|  | HCO2198 | TAAACTTCAGGGTGACCAAAAAATCA |
| 16S rDNA | RbF | GCTCAGAACGAACGCTATC |
|  | RbR | GAAGGAAAGCATCTCTGC |
