# Supplementary material for: Comparison of bacterial diversity and abundance between sexes of Leptocybe invasa Fisher & La Salle (Hymenoptera: Eulophidae) from China
Source: PeerJ. 2020 Jan 15;8:e8411. doi: 10.7717/peerj.8411 (PMC6969552; doi:10.7717/peerj.8411)
Supplement: Table S2 [file peerj-08-8411-s002.docx]

**Additional file2: Table S2: Sex of *L. invasa* specimens in this study**

| Number of Females | 656 |
| --- | --- |
| Number of Males | 51 |
| Total | 707 |
| Sex ratio (Male: Total) (%) | 7.21 |
