# Supplementary material for: Comparison of bacterial diversity and abundance between sexes of Leptocybe invasa Fisher & La Salle (Hymenoptera: Eulophidae) from China
Source: PeerJ. 2020 Jan 15;8:e8411. doi: 10.7717/peerj.8411 (PMC6969552; doi:10.7717/peerj.8411)
Supplement: Table S3 [file peerj-08-8411-s003.docx]

**Additional file3: Table S3:**

**Basic information of high-throughput sequencing based on bacteria 16S rRNA in the *Leptocybe invasa***

| Sample | Number of  PE Reads | Number of  Raw Tags | Number of  Clean Tags | Number of  Effect Tags | Effective Proportion (%) |
| --- | --- | --- | --- | --- | --- |
| Female | 169818 | 162586 | 147688 | 147402 | 86.80 |
| Male | 382629 | 370680 | 355543 | 328833 | 85.94 |
